# Preliminary Schools for Probationers

**Published:** 1909-03-20

**Authors:** 


### Nursing Administration.

### PRELIMINARY SCHOOLS FOR PROBATIONERS.

### II.—STAFF AND ACCOMMODATION.

The provision of a separate building for the preliminary school inevitably leads to serious expense. It means mounting an establishment, paying extra rent and taxes, and a very considerable outlay on maintenance. The expense of Tredegar House, the finely appointed preliminary school in connection with the London Hospital, is largely in excess of the fees paid by the pupils, and it is a commentary on the enormous advantages reaped from the preparatory course that the hospital authorities find it pays to incur this expense rather than introduce wholly untrained women into the wards. Most managers, however, would find it impossible to spend additional sums on preparation for entering the nurse-training school, and we hope to show that with some contrivance and by means of exact organisation the preparation can be given at cost price within the walls of the nurses' home.

All authorities seem to be agreed that to be of real service in preparing probationers for their future duties the preliminary instruction must be residential. It is true that at the Glasgow Royal Infirmary preliminary courses of instruction are made obligatory on the probationer, the whole series, comprising no less than 106 lectures and demonstrations, occupying a period of three months, during which the student provides board and lodging at her own expense. But this is an entirely exceptional arrangement. If copied in England, a residential college would naturally spring up for the use of the pupils, and the expense would probably be prohibitive. It is one of the most fertile sources of breakdown among probationers that they are unaccustomed to early rising and to the sensation of working close to a time-table from morning to night. They are compelled to accommodate themselves to exact routine of a somewhat arduous nature at the very moment when they are over-stimulated by having to fill a part in the hospital which they know themselves incapable of filling worthily, and the double strain is too much. The course of preliminary discipline supplied before entering the training school tides over this crisis and helps to "break in" the probationer without the distress often felt most keenly by those who subsequently make the best nurses. It may be taken, then, that sleeping accommodation must be provided for the pupils either in separate bedrooms, which is undoubtedly best, or in cubicles. It is very desirable to secure a separate wing for the accommodation of the pupils, so that they may be entirely dissociated from the ordinary staff, but this is not an actual necessity. The only other rooms required by the members of the school are a class-kitchen, and a classroom and sitting-room combined. At Guy's, where the pupils number 15, a separate sitting-room is provided, but is in practice little used.

The class-kitchen is a most important part of the equipment. It ought to be fitted up under the direction of a trained cookery instructor, and if the advice of the instructor who is to give the cookery courses can be obtained subsequent expense and alterations will be avoided. Only the simplest utensils will be required, with a gas range; but as a good part of the success of the teaching depends on proper appliances and accommodation for the pupils, it is better to avoid makeshifts as far as may be. If space allows, it is good to have a separate dining-room for the pupils; but where space is a consideration, and the number of pupils is not large, it will suffice to reserve a separate table for their use in the common dining-room. In this way only two extra rooms besides the sleeping apartments will be needed. In addition to this, however, accommodation must be found for the staff of the school. This may be brought down to very small proportions. At Guy's Hospital there is a sister in charge with a sister to relieve and take holidays, etc., and there are two maids. But in a school with fewer pupils the sister in charge could be relieved by one of the staff nurses from the hospital, who would only need to put in part time at the school. And with fewer pupils, also, one maid would be sufficient. As regards the staff, the sister in charge is the hinge on which the establishment turns. Its entire success will depend on her skill in reading character, in maintaining discipline, and imparting both tone and instruction to her pupils. It is an intensely interesting work to a woman with a talent for teaching and the true educational instinct. Many sisters possess it, but have no time to give it full play. To hold such a post for a few years would be to receive valuable preparation for the post of matron, and the hospital affords few more influential appointments. For the impetus given to the pupil to exercise herself in self-control and concentration on duty will, under congenial circumstances, last right on through the nurse's career. It is grievous, indeed, to reflect on the many good resolutions, and the genuine enthusiasm wasted in the first few weeks of hospital life owing to the fact that no one has leisure to direct them into a channel of permanent usefulness. It is desirable for the entire instruction of the pupils to devolve on the sister in charge, with the exception, perhaps, of the cookery course, for which a paid teacher may more conveniently be engaged. She should have one or two maids as required to do the rough work of the pupils' wing, the ordinary housework being done under supervision by the pupils themselves as part of their course. It will be seen that neither in staff nor in accommodation are the requirements of the preliminary school very exacting. If one floor of the Nurses' Home can be appropriated for this purpose, so much the better. But excellent results can be obtained under a less perfect organisation, and good discipline will make itself felt under many obvious drawbacks. As all the pupils are merely birds of passage the plainest furniture, provided all be neat, will suffice for the school, the sister in charge having her own sitting-room.

